# Fulminant Wilson's Disease Managed with Plasmapheresis as a Bridge to Liver Transplant

**DOI:** 10.1155/2014/672985

**Published:** 2014-09-09

**Authors:** Talal Hilal, R. Scott Morehead

**Affiliations:** ^1^Department of Internal Medicine, University of Kentucky College of Medicine, Charles T. Wethington Building 304B, 900 South Limestone Street, Lexington, KY 40536, USA; ^2^Department of Pulmonary, Critical Care and Sleep Medicine, University of Kentucky College of Medicine, Kentucky Clinic L543, 740 South Limestone Street, Lexington, KY 40536, USA

## Abstract

New-onset jaundice can be a manifestation of multiple pathologic processes including hemolysis, parenchymal liver disease, and cholestasis; the differential diagnosis is broad and requires a systematic approach. We report a case of a patient who presented with jaundice after starting minocycline for the treatment of acne vulgaris and rapidly developed fulminant liver failure found to be due to Wilson's disease. She also manifested severe Coomb's negative hemolytic anemia and renal failure secondary to hepatorenal syndrome. As a bridge to liver transplant, she was successfully treated with plasmapheresis to decrease serum copper in addition to hemodialysis for acidosis and hyperkalemia. She was able to receive a liver and made a full recovery. The case highlights the use of plasmapheresis as an adjunctive treatment modality in cases of fulminant liver failure due to Wilson's disease.

## 1. Introduction

Wilson's disease is a rare hereditary disorder of copper metabolism characterized by accumulation of copper in vital organs such as the brain and liver, among others. In rare occasions, the disease can present with fulminant liver failure that is fatal without an emergent liver transplant. We report a case of acute Wilson's disease that rapidly progressed to life-threatening multiorgan failure and discuss the presentation, diagnosis, and acute management options that can bridge patients to liver transplantation.

## 2. Case Report

A 19-year-old female presented with a two-week history of worsening scleral icterus, abdominal distension, and fatigue. She was otherwise healthy, apart from a diagnosis of acne vulgaris for which she was recently prescribed minocycline. She denied high-risk behaviors and had no family history of liver disease. On examination, the patient was afebrile, her blood pressure was 119/79 mmHg, heart rate was 105 beats per minute, respiratory rate was 20 breaths per minute, and her oxygen saturation was 99% on ambient air. She was alert and oriented with icteric sclera, a mildly distended abdomen without shifting dullness, and 1+ pitting edema of the lower extremities.

Laboratory initially revealed mildly elevated transaminases with increased bilirubin, hypoalbuminemia, and elevated international normalized ratio (INR) indicative of hepatocellular liver injury with impaired liver synthetic function (see Table 1). Hemolysis labs including haptoglobin, direct Coomb's test, and lactate dehydrogenase (LDH) were normal. An abdominal ultrasound revealed mild ascites but was otherwise normal. She was initially diagnosed with acute hepatitis of unknown etiology, with the differential diagnosis including viral hepatitis, drug-induced liver disease since she had recently started minocycline, autoimmune hepatitis, and Wilson's disease.

Further testing revealed negative results for hepatitis A (IgM and IgG), hepatitis B (surface antigen, e antigen, and core IgG), hepatitis C (IgM and IgG), anti-nuclear antibody, and anti-smooth muscle antibody, making viral and autoimmune disease extremely unlikely. Her serum ceruloplasmin level was low at 17 mg/dL (normal 22–50 mg/dL), with serum and urine copper levels of 273 mcg/dL (normal, 70–175 mcg/dL) and 8719 mcg/day (normal, 15–60 mcg/day), respectively. The findings of acute hepatitis and low ceruloplasmin level prompted a slit-lamp ophthalmologic exam revealing copper deposition in Descemet's membrane of the cornea consistent with the Kayser-Fleischer (KF) ring, confirming the diagnosis of Wilson's disease on day 4.

Clinical deterioration ensued with increasing abdomen distention and development of hepatic encephalopathy. Severe hemolysis developed on day 5 with nadir hemoglobin of 4.2 g/dL, high LDH, undetectable haptoglobin, and negative direct Coomb's test, in addition to worsening renal and hepatic function (see Table 1). The patient was subsequently transferred to the intensive care unit, placed on mechanical ventilation, and started on renal replacement therapy in the form of continuous veno-venous hemofiltration (CVVH). Plasmapheresis using fresh frozen plasma (FFP) was initiated to reduce the high serum copper level that was believed to be producing hemolysis and renal tubular damage.

She received three treatments of plasmapheresis removing a total of approximately 7,000 micrograms of copper, in addition to transfusion for anemia. Her laboratory values improved by the end of the 48-hour period prior to liver transplant (see Table 1). Orthotopic liver transplant was performed on day 7 of hospitalization. The explanted liver grossly had a tan-yellow hue with nodularity (Figure 2(a)). Histologic findings revealed chronic inflammatory changes and extensive bridging fibrosis (Figure 2(b)) with numerous Mallory bodies (Figure 2(c)). A tissue copper quantification test showed a level of 1118 mcg/g dry weight (normal 10–35 mcg/g).

Her hepatic function tests improved and her kidney function normalized. She was discharged on standard immunosuppressive therapy but is expected to have no further complications related to copper overload.

## 3. Discussion

Wilson's disease is a rare autosomal recessive genetic disorder of copper metabolism characterized by numerous mutations in the ATP7B gene on chromosome 13. The gene encodes a transmembrane protein ATPase that functions to transport copper outside the cell, incorporate it as a copper-ceruloplasmin complex, and excrete excess copper in bile. A mutation in the ATP7B protein causes cytoplasmic copper accumulation in many organs including the cornea, brain, and liver cells [1]. Wilson's disease affects 1 in 30,000–100,000 individuals and usually presents in the second to third decades of life. Clinical manifestations are mainly neurologic, psychiatric, and/or hepatic. Patients who present with neuropsychiatric symptoms (e.g., depression, neurosis, tremor, choreiform movements, and/or seizures) tend to be older, and the concurrent liver disease is often felt to be unrelated and incidental. By contrast, the present patient's presentation with fulminant liver failure and no neuropsychiatric findings occurs in only 5% of cases [2].

**Figure 1:**
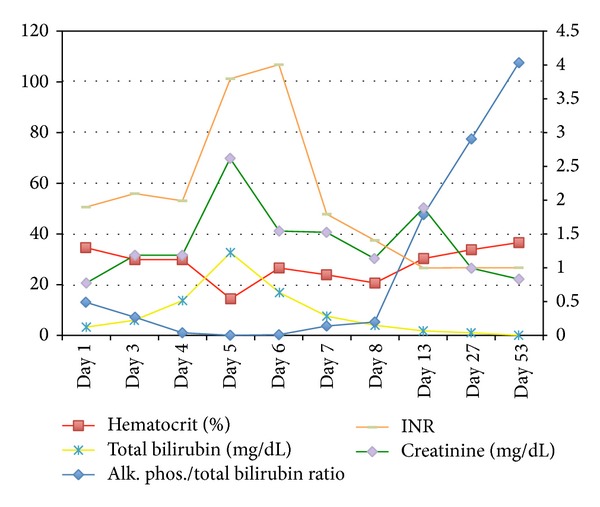
Trend of lab values during the acute setting of liver failure and posttransplant up to a 2-month follow-up.

**Figure 2:**
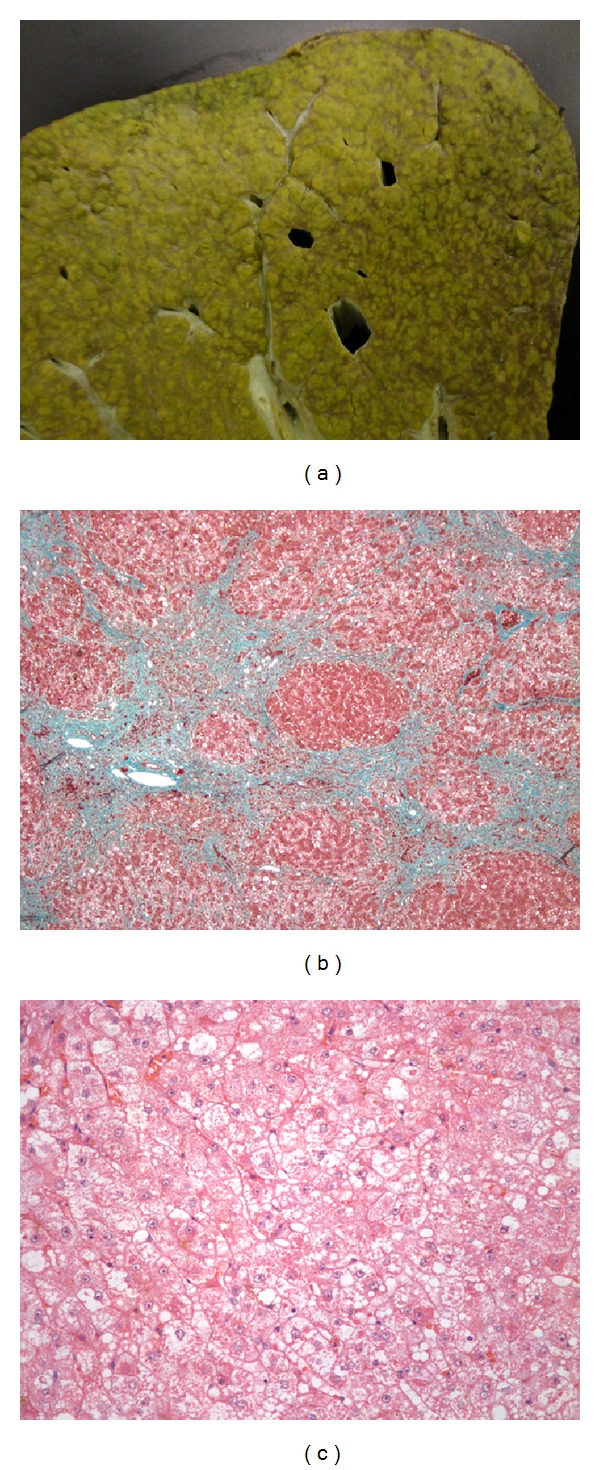
(a) Gross image of explanted liver with a nodular, tan-yellow surface. (b) Histologic section of liver tissue depicting extensive bridging fibrosis. (c) Histologic section depicting ballooning degeneration and numerous Mallory hyalines indicative of chronic disease.

The diagnosis of Wilson's disease can be difficult due to the variability in clinical manifestations and time course. Multiple diagnostic scoring systems have been developed but none have been prospectively validated. Using the system developed by Ferenci et al. [3], the findings of KF rings, low ceruloplasmin level, Coomb's negative hemolytic anemia (with high serum copper), urinary copper > 2x ULN, and liver copper quantitative > 5x ULN made the diagnosis of Wilson's disease “highly likely,” with a score of 8 out of a possible 16. In the acute setting, clinical suspicion (e.g., age of patient, family history, KF rings, and low ceruloplasmin level) should guide initiation of therapy before fulminant liver failure ensues. Furthermore, the finding of a ratio of alkaline phosphatase concentration to total bilirubin concentration of <2 was identified by Berman et al. [4] in 1991 as providing 100% sensitivity and specificity for fulminant Wilson's disease (present in this patient on day 4—see Table 1 and Figure 1). The validity of this index has not been confirmed by other studies [5].

Circulating copper is normally loosely bound and transported by albumin, constituting the free copper in healthy subjects that is greatly elevated in patients with fulminant liver failure due to Wilson's disease [6]. The specific trigger for fulminant liver failure is not known, but elevated serum copper damages both RBC membranes with resultant intravascular hemolysis and renal tubular cells causing renal failure. Multiple methods have been used to reduce the copper load in the acute setting with varied clinical results. Plasmapheresis with FFP replacement has shown to be efficacious in rapidly reducing serum copper levels [7]; other methods that have been used include dialysis, albumin dialysis, and molecular adsorbents recirculating system (MARS) [8–11]. The chelating agents D-penicillamine and trientine have been used to promote renal copper excretion in stable Wilson's disease patients; however, with fulminant disease and rapidly progressive renal failure, the benefit is unclear. There have been reports of the use of D-penicillamine in combination with plasmapheresis in the acute setting with successful outcomes [10]. Nonetheless, the mortality rate in patients with fulminant liver failure approaches 100% without emergent liver transplant.

Liver transplantation is indicated for patients with Wilson's disease in the setting of fulminant liver failure or chronic liver disease unresponsive to medical management. Liver transplantation results for Wilson's disease appear to be excellent with multiple studies reporting 5-year survivals >85% and excellent long-term prognosis [11–13]. The largest of these series was reported from France, including 121 patients transplanted for Wilson's disease between 1985 and 2009, with the reported survival rate of 87% at 5, 10, and 15 years following transplantation [14].

## 4. Conclusion

Wilson's disease should be suspected in young patients presenting with fulminant liver failure of unknown etiology. In this setting, plasmapheresis is effective for stabilization of clinical and laboratory parameters and served as a bridge to liver transplant. This case further supports the use of serum copper-reducing modalities in the setting of fulminant Wilson's disease.

## Figures and Tables

**Table 1:** Trend of laboratory values before and after liver transplant.

	Day 1	Day 3	Day 5	Day 6	Day 7 (transplant)	Day 8	Day 13	Day 27	Day 53
Hemoglobin (11.2–15.7 g/dL)	11.2	9.6	4.2	9.1	8	7.3	9.5	11.2	12.4
Hematocrit (34–45%)	34.9	30	14.8	26.8	23.8	20.8	30.4	34.1	36.8
MCV (79–98 fL)	99	101	131	96	93	89	91	93	90
Platelet (155–369 k/uL)	202	166	162	88	50	32	123	320	238
WBC (3.7–10.3 k/uL)	15.9	14.2	38.1	19.2	7.6	6.4	9.7	11.5	9.7
BUN (7–21 mg/dL)	15	19	41	18	17	15	33	28	19
Creatinine (0.60–1.10 mg/dL)	0.78	1.2	2.63	1.54	1.52	1.13	1.89	1	0.84
Sodium (136–145 mmol/L)	135	136	134	141	143	140	139	140	138
Potassium (3.7–4.8 mmol/L)	4.5	4.3	5.6	4.3	3.9	3.4	3.5	4.3	3.8
AST (11–32 U/L)	159	150	187	323	626	347	57	41	16
ALT (8–33 U/L)	89	19	11	230	518	340	122	49	32
Alkaline phosphatase (52–144 U/L)	47	44	<5	6	28	22	85	70	43
Total bilirubin (0.2–1.1 mg/dL)	3.4	6.4	32.9	17.2	7.6	4	1.8	0.9	0.4
Albumin (3.3–4.6 g/dL)	2.1	2	3.2	2.7	2.4	2.4	2.2	3.7	4.1
INR	1.9	2.1	3.8	4.8	1.8	1.4	1	1	1
